# Gamma-Retrovirus Integration Marks Cell Type-Specific Cancer Genes: A Novel Profiling Tool in Cancer Genomics

**DOI:** 10.1371/journal.pone.0154070

**Published:** 2016-04-20

**Authors:** Kathryn L. Gilroy, Anne Terry, Asif Naseer, Jeroen de Ridder, Amin Allahyar, Weiwei Wang, Eric Carpenter, Andrew Mason, Gane K-S. Wong, Ewan R. Cameron, Anna Kilbey, James C. Neil

**Affiliations:** 1 MRC-University of Glasgow Centre for Virus Research, Institute of Infection, Immunity and Inflammation, College of Medical, Veterinary and Life Sciences, University of Glasgow, Glasgow, United Kingdom; 2 Delft Bioinformatics Lab, Faculty of EEMCS, Delft University of Technology, Delft, The Netherlands; 3 Department of Biological Sciences, University of Alberta, Edmonton, Alberta, Canada; 4 Centre of Excellence for Gastrointestinal Inflammation and Immunity Research, University of Alberta, Edmonton, Alberta, Canada; Texas A&M University, UNITED STATES

## Abstract

Retroviruses have been foundational in cancer research since early studies identified proto-oncogenes as targets for insertional mutagenesis. Integration of murine gamma-retroviruses into the host genome favours promoters and enhancers and entails interaction of viral integrase with host BET/bromodomain factors. We report that this integration pattern is conserved in feline leukaemia virus (FeLV), a gamma-retrovirus that infects many human cell types. Analysis of FeLV insertion sites in the MCF-7 mammary carcinoma cell line revealed strong bias towards active chromatin marks with no evidence of significant post-integration growth selection. The most prominent FeLV integration targets had little overlap with the most abundantly expressed transcripts, but were strongly enriched for annotated cancer genes. A meta-analysis based on several gamma-retrovirus integration profiling (GRIP) studies in human cells (CD34+, K562, HepG2) revealed a similar cancer gene bias but also remarkable cell-type specificity, with prominent exceptions including a universal integration hotspot at the long non-coding RNA *MALAT1*. Comparison of GRIP targets with databases of super-enhancers from the same cell lines showed that these have only limited overlap and that GRIP provides unique insights into the upstream drivers of cell growth. These observations elucidate the oncogenic potency of the gamma-retroviruses and support the wider application of GRIP to identify the genes and growth regulatory circuits that drive distinct cancer types.

## Introduction

The ability of retroviruses to direct stable integration is inherently mutagenic and can perturb host cell genes at the proviral insertion site or even at a significant distance [1,2]. While this feature has come to be regarded mainly as an unwanted complication for the use of retroviral vectors in gene therapy [3,4], it has long been a valuable asset in cancer research, as the integration sites of retroviruses in naturally occurring or experimentally induced cancers from birds, mice and cats have provided a rich harvest of cancer driver genes and families including *Myc*, *Myb*, *Pim*, *Runx*, *Bmi1*, *Gfi1* and *Notch* [1]. The completion of the mouse genome sequence and the advent of high throughput cloning and sequencing of insertion sites expanded the scope of these studies massively, revealing many new potential target genes [5–7]. It was assumed in early studies that retroviral integration is effectively random, and that cancer-specific common integration sites (CISs) arose merely from clonal expansion after rare insertions at sensitive sites that coincided in independent tumours by chance. In contrast, it has become clear in recent years that retroviruses have significant integration preferences that reflect their distinctive biological features and modes of host colonisation.

The predilection of the lentivirus HIV to integrate into actively transcribed genes is a key feature of its pathogenesis where it transits between latent infection and cytopathic replication. For HIV, this process entails interaction between the viral integrase protein and LEDGF, a transcriptional co-activator that tethers the integration complex to chromatin and facilitates integration [8,9]. In contrast, gamma-retrovirus replication is generally non-cytopathic and persistently infected hosts can display high levels of viraemia, with tumours induced by insertional mutagenesis a relatively common outcome of infection [10,11]. The non-randomness of murine leukemia virus (MLV) integration was first appreciated from early studies that highlighted biases towards DNaseI hypersensitive sites and transcriptional start sites [12]. The basis of this specificity was recently elucidated with the demonstration that the MLV integrase interacts with BET/bromodomain proteins (Brd2, 3 and 4) that in turn bind to acetylated histone H3K27ac, marking some of the most active regions of chromatin [13–15]. The importance of this interaction is underlined by the significant reduction in titre and/or loss of TSS targeting of MLV grown in the presence of BET inhibitors JQ1 and I-BET *in vitro* [13–15]. This creates a further connection with cancer research as BET inhibitors are currently being investigated in clinical trials for the treatment of multiple cancers [16].

The full extent of departure from random of MLV integration has become clear only with the advent of large scale methods to capture and sequence integration sites in polyclonally infected cell populations *in vitro* prior to any significant growth selection. Large scale studies of MLV vector integration in human CD34 cells or MLV pseudotype infection of human cancer cell lines has revealed a remarkably selective process in which more than half of the integrations target less than 2% of the human genome [17,18]. Moreover, the preferred genomic sites occur at active chromatin marks and include strong enhancers as well as promoters.

In this study we explored the integration preferences of another gamma-retrovirus, feline leukaemia virus (FeLV). We used FeLV-B, a common naturally occurring variant of FeLV that is capable of infecting virtually all cultured human cells without evident cytopathology [19] through interaction with the widely expressed phosphate transporter PIT1 [20]. We initially analysed integrations in the MCF-7 human breast cancer cell line which is permissive for spreading, high titre FeLV-B replication, and is among the best characterised cancer cell lines with respect to functional genomics. FeLV-B displayed a similar preference for transcription start sites and active chromatin marks to MLV, consistent with conservation of the C-terminal loop of integrase that binds to BET/Brd [21]. However, FeLV integration specificity was not primarily directed to the most abundantly expressed genes but was strongly skewed towards breast cancer driver genes. This finding inspired a meta-analysis of gamma-retrovirus integration preference from several studies which revealed a high degree of cell-type specificity, while cancer genes were favoured in all cases, including in normal cells. These findings suggest that gamma retroviral integration profiling (GRIP) will be a valuable tool for the identification of lineage-specific cancer driver genes in a wide variety of human cancer types.

## Results

### FeLV integration in human breast cancer cells targets transcription start sites and active chromatin marks

**Fig 1:**
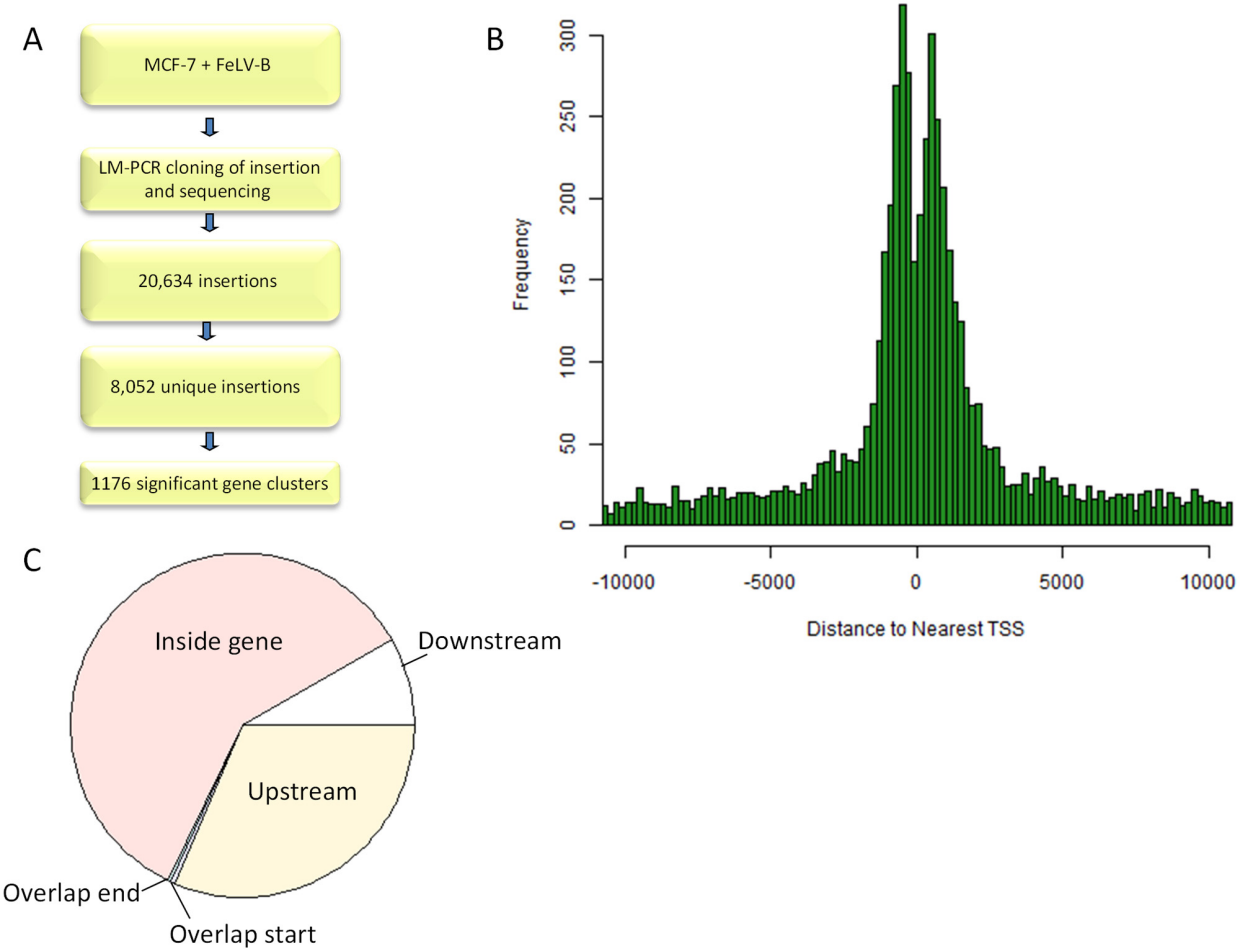
A: Experimental workflow. B: Position of insertions in MCF-7 cells with respect to TSS, showing a double peak with trough at TSS. C: Pie chart showing position of insertions in MCF-7 cells with respect to the nearest gene.

Cloning of FeLV-B integration sites in infected MCF-7 breast cancer cells by linker-mediated PCR and mapping to the human genome yielded 20,634 authentic virus-host junction fragments, corresponding to 8,052 unique insertions (Fig 1A, S1 Dataset). The relatively low number of copies per unique insertion suggested that no significant clonal selection had occurred during the brief period of growth *in vitro*, and this question was examined further by analysis of proviral orientation bias. While the integration process itself is random with regard to orientation, the propensity of gamma-retroviruses to activate host genes by enhancer insertion, a process which is strongly affected by orientation, leads to emergence of dominant clones with pronounced bias at key integration hot-spots that can be demonstrated statistically by a ‘heads-tails’ analysis [22]. We applied this test to the FeLV/MCF-7 dataset. Of the 100 genes most frequently targeted, 8 showed evidence of orientation bias (p values 0.011–0.049 by Fisher’s exact test), but none of these observations survived Bonferroni or Benjamini-Hockberg correction for multiple testing (in all 8 cases p value with Bonferroni correction was 1, and p value with Benjamini-Hockberg correction was 0.612). Moreover, none of the nearby genes were annotated cancer drivers, and none displayed the classic ‘upstream and backwards’ clustering that is most frequently observed with this mode of oncogene activation [1]. Additionally, insertions at the 100 most frequently targeted genes showed no evidence of increased copy number compared to the dataset as a whole, with an average of 2.7 and 2.6 copies/insertion respectively (p = 0.44). These observations suggest that minimal clonal selection has occurred after integration and that any observed non-random distribution in the genome is due principally to insertion site preference. Clustering of FeLV insertions around transcription starts sites (TSSs) was evident from the dataset, with a double peak at +/-1.5kb and a trough directly at the TSS indistinguishable from the pattern reported for MLV [17] (Fig 1B). The position of the insertions within the nearest gene was determined and is shown in Fig 1C. Most of the insertions lie inside the gene, with the next largest group upstream of the gene, consistent with their targeting of enhancer elements.

**Fig 2:**
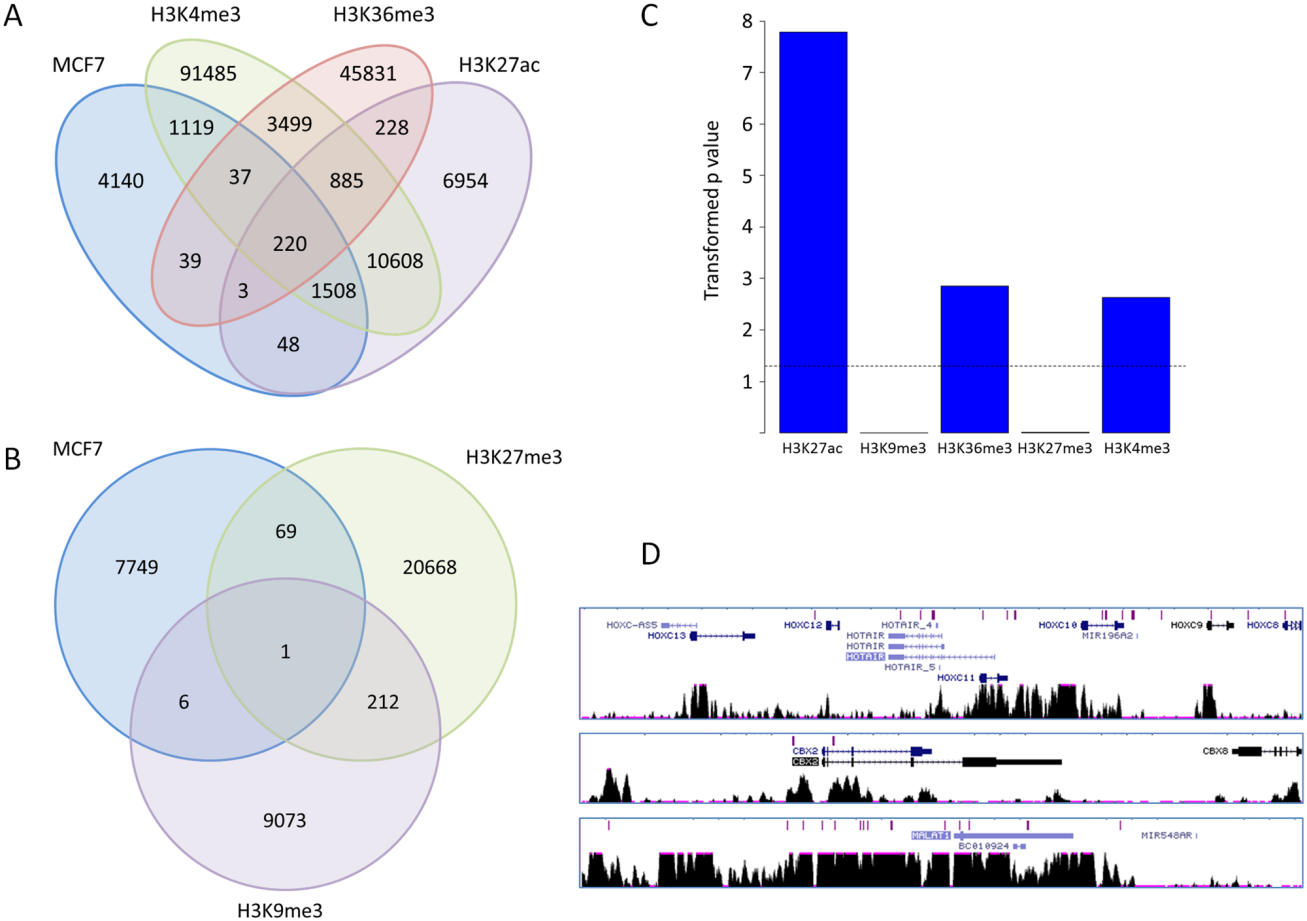
Association of MCF-7 insertions with histone marks. A: Global association of insertions with active histone marks showing the number of intersecting features. B: Global association with repressive histone marks. C: Statistical association of the most significantly enriched nearest genes for insertions and histone marks. Transformed p values are shown. The dotted line represents p = 0.05. D: Example gene clusters targeted by FeLV in MCF-7 cells, as displayed in the UCSC Genome Browser. Insertions are shown in purple at the top, followed by schematics of gene structure, and finally H3K27ac ChIP-seq signal density.

To determine whether specific epigenetic marks were also targeted by the virus, data from the ENCODE consortium were analysed. ChIP-seq datasets for all available histone marks in MCF-7 cells were processed and peaks annotated as described in Materials and Methods. Available histone marks were H3K27ac, H3K9me3, H3K36me3, H3K27me3 and H3K4me3. The overlap of MCF-7 insertions with histone marks was determined and is summarised in Fig 2. Firstly markers of active chromatin were considered, these being H3K27ac (enhancer mark), H3K4me3 (active promoter mark) and H3K36me3 (marker of elongation). As summarised in Fig 2A, a large proportion of the MCF-7 insertions overlap with these histone marks (41.8%); the largest overlap occurs with either H3K4me3 alone (1119 insertions, 15.7%) or with both H3K4me3 and H3K27ac (1508 insertions, 21%). When considering repressive marks H3K9me3 and H3K27me3, there was very little overlap with MCF-7 insertions (just 0.97% in total, Fig 2B). Considering the significance of pairwise overlap of MCF-7 insertions with each of the five histone marks, none were statistically significant with the exception of H3K27ac, which was highly significant with a p value of 4.96E-69 (p values for all other pairwise overlaps were 1). Having examined the global relationship between histone mark annotation and MCF-7 insertion, the relationship to histone marks of the most significant insertion clusters was examined. A ‘nearest gene’ analysis was performed as described in Materials and Methods, assigning each insertion to a gene and assessing the significance of the insertion at that gene. Similarly, histone marks were annotated and significance scores (p-scores) obtained using the Galaxy/Cistrome suite as described in Materials and Methods. The top 500 gene hits for each histone mark (identified by p-score) were collated and compared to the top retroviral integration targets and overlap assessed. The likelihood of such an overlap occurring by chance was assessed using Monte-Carlo simulation as described in Materials and Methods. Data are summarised in Fig 2C and Table 1. The three active chromatin marks show significant overlap when the most significant genes clusters are considered, with p values of <1.67E-8, 0.00236 and 0.0014 for H3K27ac, H3K4me3 and H3K36me3 respectively. The repressive chromatin marks H3K9me3 and H3K27me3 show no significant association with MCF-7 insertions (p = 1 and p = 0.97 respectively). Overall, comparison with all available histone modifications shows consistently that insertions are targeted to active chromatin marks and are absent from repressive marks.

**Table 1:** Statistical association of the most significantly enriched nearest genes for insertions and histone marks. The number of overlapping genes from the top 500 are noted, as are the p values for significance of overlap. Note that the p value for H3K27ac represents a 1 in 60x10^6^ chance of the association occurring by chance, but the true p value will be lower as this level of overlap was not observed in 60x10^6^ simulations.

Histone mark	Number of Overlapping Genes	p value	Transformed p value
H3K27Ac	56	1.67E-08	7.77815125
H3K9me3	1	0.999999	4.34E-07
H3K36me3	76	0.001402	2.853251986
H3K27me3	6	0.970586	0.012965977
H3K4me3	13	0.002363	2.626536278

### FeLV integration targets cancer driver genes in MCF-7 breast cancer cells

Previous large scale studies have focused on murine gamma-retrovirus and vector integration in normal and malignant cells and it was noted anecdotally that many vector insertions in normal CD34 cells were at ‘dangerous’ sites with regard to the risk of malignancy, including the LMO2 locus which has featured as a frequent target of vector integration in gene therapy associated leukaemias [3,4]. Initial inspection of the major clusters of FeLV insertion into MCF-7 cells showed a remarkable concentration at genes with reported over-expression or amplification in breast cancer or with a loss-of-function knockdown phenotype in MCF-7 cells. Example gene clusters shown in Fig 2D include three HOX gene clusters, including the long non-coding RNA *HOTAIR*, along with chromobox *CBX2* and the long non-coding RNA *MALAT1*. In most cases the insertions coincided primarily with peaks of H3K27 acetylation, and to a lesser extent with the H3K4 trimethylation and H3K36 trimethylation marks, consistent with data above showing association with active chromatin marks (full histone annotation for these genes is shown in S1 Fig).

These observations encouraged us to carry out a more systematic analysis of the preferred insertion sites. Nearest gene analysis identified 6926 genes. The top 100 genes were determined by first selecting for statistical significance (p<0.05 using Fisher’s Exact test), and then ranking by order of the number of insertions. The top 100 MCF-7 integration targets by these criteria encompass 773 insertions (6.7% of total insertions), and are shown in S1 Table. These were cross-checked against the Cancer Gene Census [23], a stringent, regularly updated database of cancer genes based on strong evidence of driver gene status (http://cancer.sanger.ac.uk/census/).

**Fig 3:**
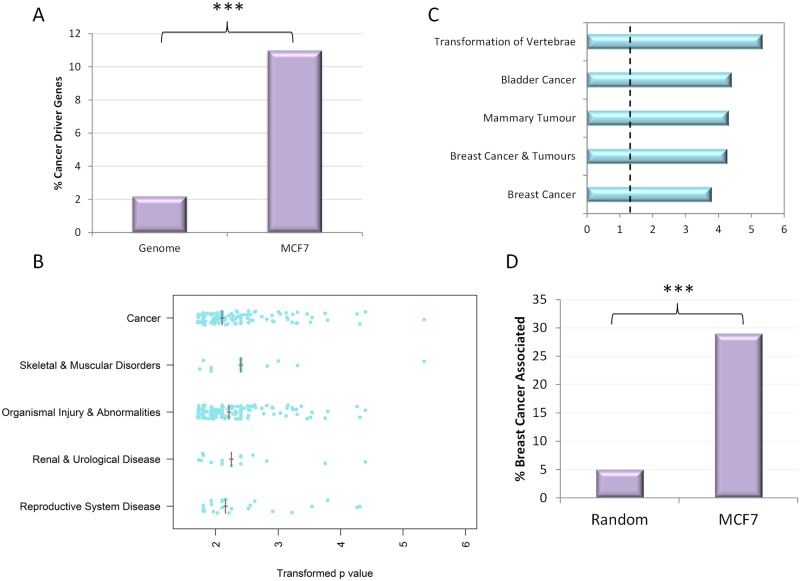
MCF-7 cancer association. A: Percentage of top 100 insertion gene clusters that are cancer driver genes compared to the genome as a whole. B: IPA top processes groupings showing the range of transformed p values, with median indicated by grey cross. C: Top 5 ‘cancer’ subtypes identified by IPA with transformed p value shown. Dotted line represents p = 0.05. D: Percentage of the top 100 insertion gene clusters with known breast cancer annotation as determined by systematic literature review compared to a random set of 100 genes.

11 of the top 100 MCF-7 integration targets were found to be cancer driver genes (see Fig 3A and S2 Table). This is a highly significant enrichment over the background level of cancer driver genes in the genome as a whole, which is 2.2% (p = 2.01E-09). To investigate the pathways targeted by FeLV integration, the top 100 target genes were interrogated by the QIAGEN Ingenuity Pathway Analysis (IPA) software suite (IPA, QIAGEN Redwood City, www.qiagen.com/Ingenuity). We found that the top biological process defined for this gene set was ‘Cancer’ (p value range 1.95E-2–4.6E-6, median p = 0.00789), providing further evidence of selectivity for cancer gene programmes (Fig 3B). Having established preferential targeting of cancer driver genes, the cell type specificity of target genes was examined. IPA ‘cancer’ annotation for the top MCF-7 hits was examined further by looking at cancer sub-processes. Strikingly, three of the top five cancer sub-processes were related to breast cancer, showing a strong tissue specific cancer gene signature in the top integration targets (Fig 3C).

To verify the enriched breast cancer gene annotation in the MCF-7 top integration sites, a screen of the peer-reviewed scientific literature was performed for the top 100 MCF-7 target genes compared to 100 genes chosen at random. To minimise bias, in each case the same search term was used (<gene name> + “breast cancer”), and the same criteria for known breast cancer annotation (direct association of the gene with breast cancer with subsequent verification by e.g. knockdown or expression studies). 29 of the top 100 MCF-7 integration targets had known breast cancer annotation, compared to 5 of the random 100 genes, a difference that was highly significant (p = 3.35E-28, Fig 3D). Taken together these results indicate that integration preferentially targets a cell type-specific gene set that is also relevant to the cancer phenotype.

### Genes at preferred insertion sites of FeLV display heterogeneous levels of expression

**Fig 4:**
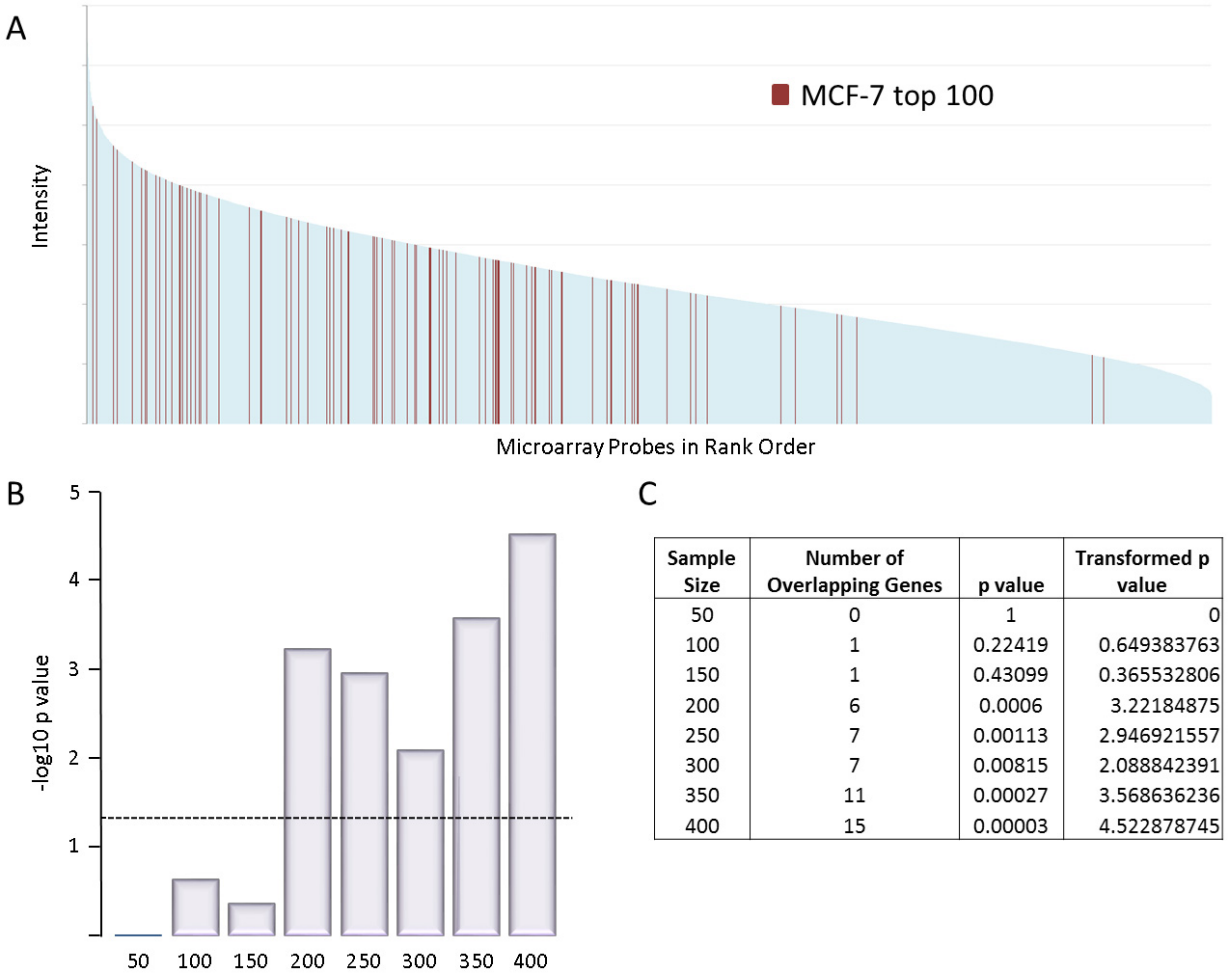
Integration preference with expression level. A: rank order plot showing intensity of all microarray probes for MCF-7. Shown in red are the top 100 gene clusters targetted by FeLV insertion. B: Transformed p values of the significance of association between the top insertion target genes and the most highly expressed genes for different sample sizes. The dashed line represents p = 0.05 significance level. C: Table showing the p values represented in B for the different sample sizes.

Previous studies have shown that gamma-retroviruses integrate into regions of active gene expression, often in regulatory regions such as enhancer and promoter regions. To determine whether FeLV targets the most highly expressed genes, the top MCF-7 integration targets were compared to the most highly expressed genes in MCF-7 cells using a previously published microarray dataset [24]. MCF-7 genes were ranked according to intensity and the position of the top 100 GRIP target genes noted (Fig 4A). This analysis revealed no apparent bias of FeLV integration for the most highly expressed genes. To analyse this relationship statistically, increasing sample sizes were used to examine how the relationship varied when considering only the top hits or a wider gene selection. The level of overlap at each sample size was compared to that predicted to occur by chance by Monte-Carlo simulation, and p values calculated (see Materials and Methods for details).

The top 150 retroviral target genes showed no greater overlap with the top 150 highly expressed genes than expected by chance in MCF-7 cells (p values for the top 50, 100 and 150 genes were 1, 0.22 and 0.43 respectively (Fig 4B and 4C)). The long non-coding RNA *MALAT1* was the only genetic element that overlapped the most highly expressed genes and the preferred retroviral targets in this analysis. With the caveat that transcription rates and steady state RNA levels are not synonymous, these results suggest that the top 150 retroviral integration targets are being selected by a more subtle process than affinity for the most active regions of host chromatin. In contrast, extending the analysis to a larger number of preferred targets (>200) detected increasing overlap with the most highly expressed genes, suggesting a bimodal selection process.

### Gamma-retroviral integration preference: a meta-analysis

Having shown that FeLV selectively targets cancer driver genes in a human breast cancer cell line, we applied the same approach to published datasets of ‘unselected’ gamma retrovirus integration sites in human cells to establish the generality of these observations. For this meta-analysis we used published insertion data from two human cancer cell lines, K562 and HepG2 [18] and normal CD34+ cells [17]. While these studies were carried out with murine gRVs and infection of human cells was achieved by pseudotyping of infectious MLV virus with VSV-G envelope [18] or amphotropic vector delivery [17] rather than natural infection, a histone code preference very similar to our study was noted, suggesting that the conserved integrase function is the major determinant of specificity.

**Fig 5:**
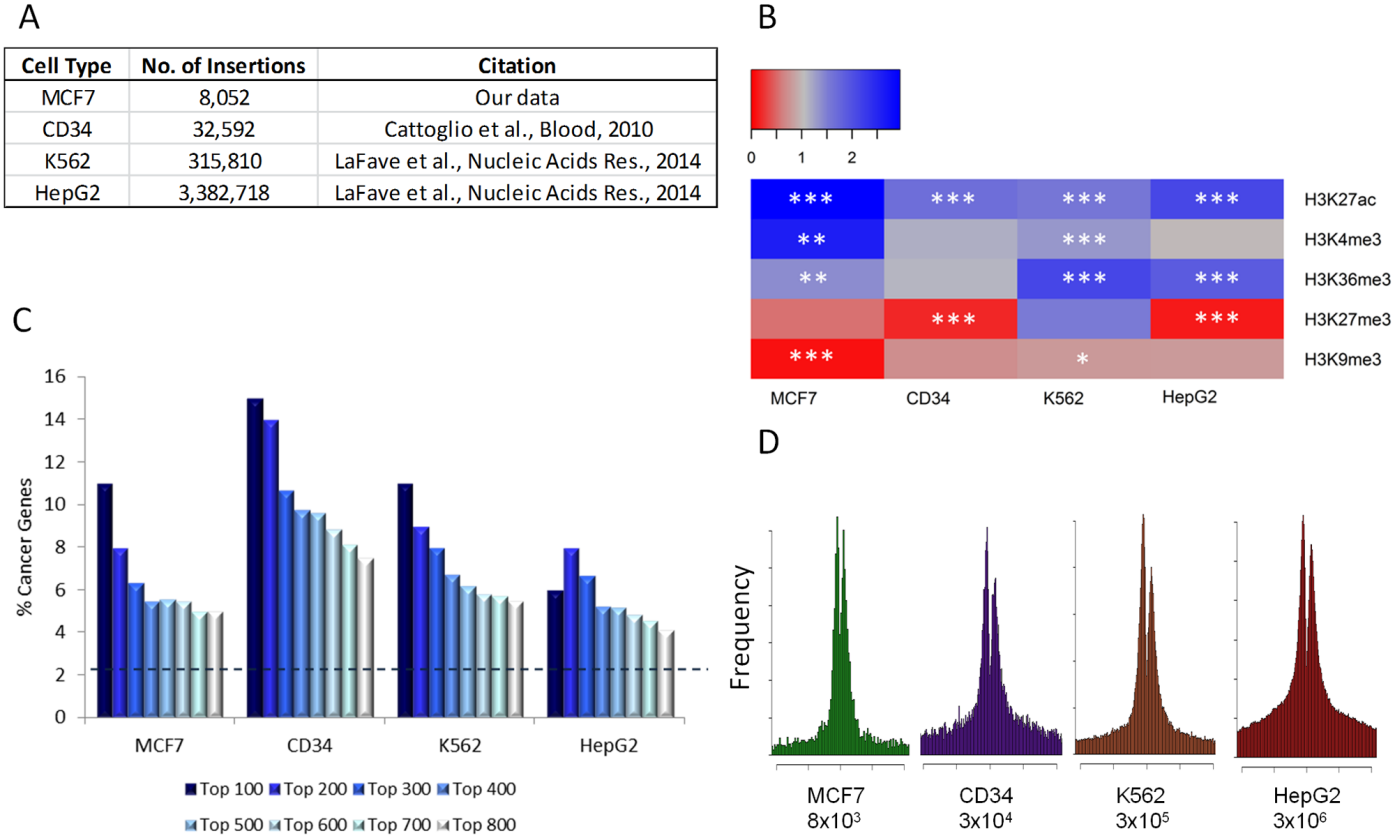
Meta-analysis of gRV gene targeting in multiple cell lines. A: Summary of the data used including the size of the datasets. B: Heatmap showing the global association of gRV insertion sites with histone modification for all cell lines. Blue represents favoured marks while red represents disfavoured marks. Asterisks denote significance level, with * being p<0.05, ** p<0.01 and *** p<0.001. C: Enrichment of cancer driver genes with refinement of top hits for all cell lines. The dashed line represents the percentage of cancer driver genes in the genome (2.2%) D: Position of insertions with respect to transcriptional start site for all cell lines. Approximate number of total insertions is shown below each chart.

Details of the datasets used in the meta-analysis are summarised in Fig 5A. The published sets are substantially larger than our MCF7 set and to obtain a similar number of preferred insertion site genes we set a higher significance threshold before ranking the genes by number of insertions as previously. For three MLV datasets there was again a highly significant enrichment for cancer driver genes. For CD34+ cells, 15% of the top target genes scored as cancer drivers (enriched compared to total genome baseline, p = 2.69E-18), for K562 the number was 11% (p = 2E-9) and for HepG2 6% (p = 0.0096).

The epigenetic marks targeted by the virus in each cellular context were examined as described above using ChIP-seq data from ENCODE (for K562 and HepG2 cells) or the Human Epigenetics Roadmap (for CD34 cells). The same histone modifications were considered as for MCF-7 cells, these being H3K27ac, H3K4me3, H3K36me3, H3K9me3 and H3K27me3. In accord with the previously published studies, there was a general enrichment for viral insertions at active chromatin marks (H3K27ac, H3K4me3 and H3K36me3) while repressive marks (H3K9me3 and H3K27me3) were disfavoured by the virus [17,18]. The only exception to this pattern was K562s which showed an enrichment of insertions at the H3K27me3 mark, although this wasn’t statistically significant. The results are summarised in Fig 5B.

Fig 5C depicts a clear trend with increasing enrichment for cancer driver genes towards the most highly targeted genes in all four cell lines. This pattern is least clear for the HepG2 dataset, although there is reason to suspect that the top hits may have been obscured by saturation in this huge dataset (3 x 10^6^), due to scoring of genuinely independent insertions at the major hotpots as duplicates. Evidence in support of this interpretation is provided by inspection of the distribution of insertions around transcriptional start sites. When these are normalised by peak height, the HepG2 shows a much larger basal level of non-TSS insertions, consistent with the saturation hypothesis (Fig 5D).

**Fig 6:**
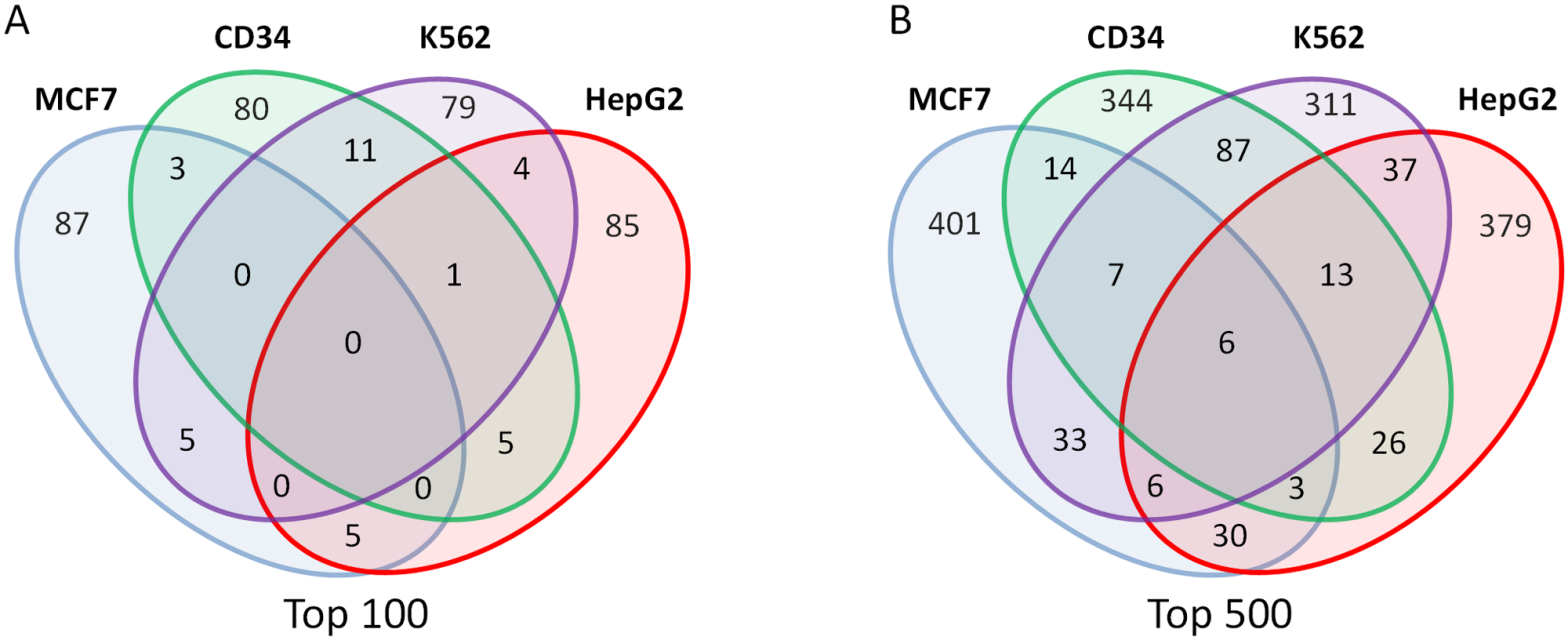
Gene comparison in multiple cell types. A: Venn diagram showing the level of overlap between the top 100 gene targets in each cell line tested. B: As (A) but considering overlap of the top 500 genes in each cell line.

Fig 6A shows the overlap of the genes in the top 100 for each of the cell lines tested. While there are some common target genes shared by more than one cell type, what is most striking is that most of the top hits are unique to that particular cell type with relatively little overlap. Expanding the datasets to encompass the top 500 genes does not diminish this generally exclusive pattern according to cell type, although this more relaxed cut-off revealed a small subset of six ‘universally’ targeted elements: *MALAT1*, *MIR7851*.*1*, *RCC1*, *VMP1*, *ZC3H4* and *ZMYND8* (Fig 6B).

### Gamma-retroviral integration profiling is complementary to super-enhancer profiling

The recent finding that gRV integration is mediated by binding to BET revealed a compelling link to cancer, as BET binding to acetylated histones is one of the defining feature of ‘super-enhancers’, a term coined to describe large clusters of enhancers that display dense binding of master regulators and play a role in tissue-specific cell identity [25]. Moreover, inhibitors of BET binding can disrupt the growth of cancer cells [16] and this phenomenon has been attributed to the acute sensitivity to BET disruption of super-enhancers at oncogenes such as MYC and BCL2 [26,27].

**Fig 7:**
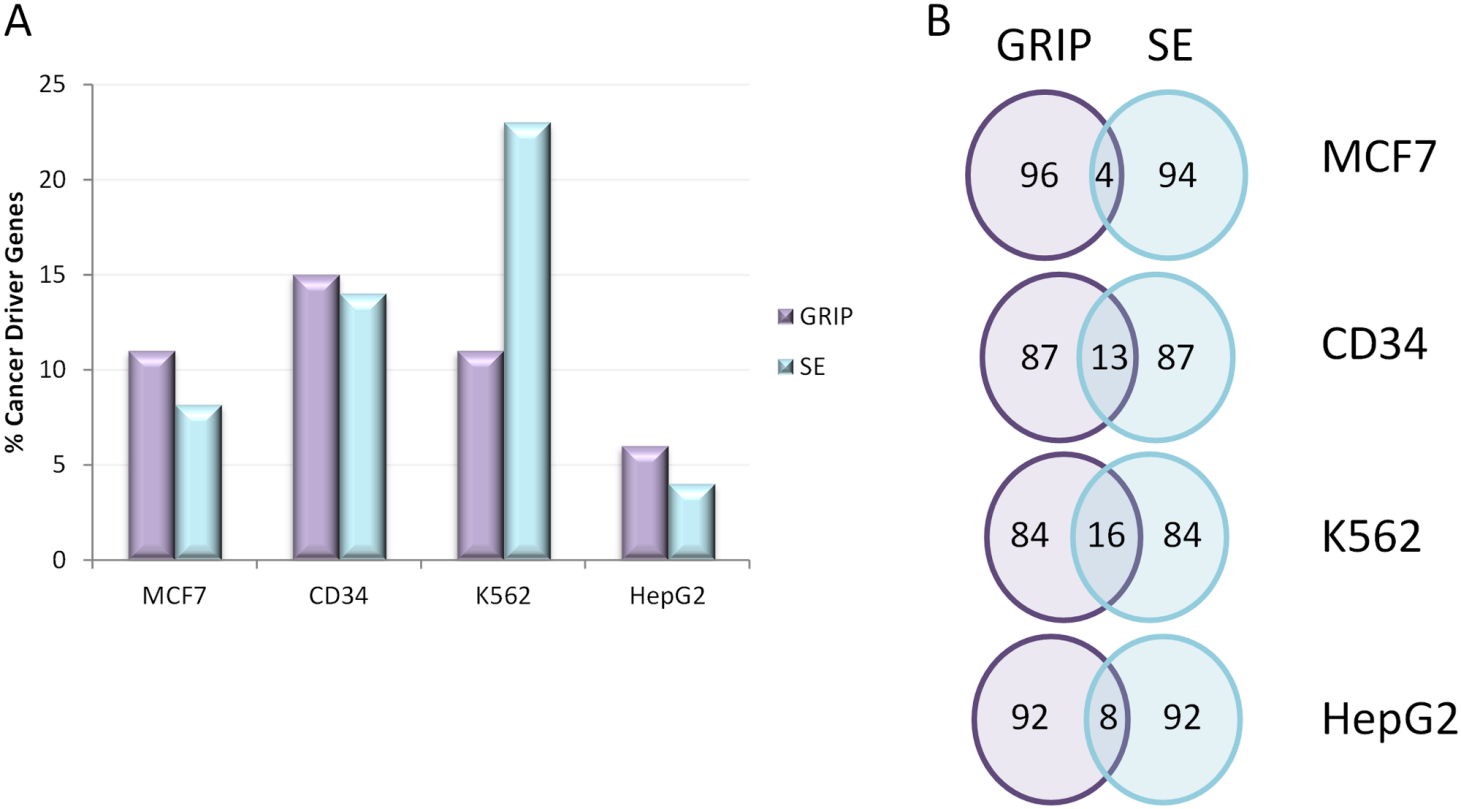
Comparison of GRIP with superenhancers. A: Percentage of the top 100 genes identified for GRIP and superenhancers that are cancer driver genes for all cell lines. B: Overlap between the top 100 genes for GRIP and superenhancers in each cell line.

To investigate the parallel between GRIP and super-enhancers defined by biochemical approaches, we compared the nearest genes identified by both methods. Lists of super-enhancer associated genes were obtained from the dbSUPER database [28] for MCF7, K562, HepG2 and CD34 primary cells (RO01480, RO01536 and RO01549). For MCF-7 cells, only 98 genes were recorded in the super-enhancer list, and of these 8 were cancer driver genes as defined by the most recent Cancer Gene Census data, compared to 11 of the top 100 GRIP targets (Fig 7A). Similar observations were noted for the other datasets, and only the K562 set revealed a stronger enrichment for cancer driver genes in the super-enhancer associated genes over the top GRIP targets (23% vs 11%; p = 0.004). The difficulty in defining a precise cut-off between super-enhancers and conventional enhancers [29] is illustrated by the large variation in the number of super-enhancers for a given cell type, with the three primary CD34+ dbSUPER entries having numbers of superenhancers ranging from 326 to 733.

Despite the similar numbers of cancer driver genes identified using the two methods, the genes identified were mostly distinct, and only 2/11 cancer driver genes identified by GRIP were also noted in the super-enhancer database for MCF-7 cells (S2 Table). Looking at the wider gene sets, there was also relatively little overlap between genes identified in the top 100 GRIP and super-enhancer targets (Fig 7B).

### Pathway analysis reveals distinct upstream regulators of retroviral targets and super-enhancer associated genes

The IPA software suite includes a module to assess the likely upstream transcriptional regulators of any abstracted gene set, helping to illuminate biological activities in that cellular system. To obtain a comprehensive and statistically robust analysis, we analysed the top 500 GRIP target genes for each cell type (Table 2). The strongly predicted upstream drivers for the MCF-7 GRIP dataset included mainly cancer drivers (MYC, KMT2A) and cancer associated genes (ATF4). Notably, these genes were themselves highly targeted by retroviral insertion, reflecting their transcriptionally active status and accessibility to integration. This analysis suggests that these genes are involved in orchestrating expression of the GRIP target set and hence may represent the critical drivers of the cancer programme. Similar observations were noted for the other cancer cell lines, and for normal CD34+ cells, although in the latter case two of the most strongly predicted upstream drivers were cytokines (CSF, IL15) which were not targeted by retroviral integration and were apparently not transcriptionally active in the target cells.

**Table 2:** Upstream regulators identified by pathway analysis for GRIP and superenhancers using the top 500 genes for each cell line.

	GRIP			Superenhancers		
	Driver Gene	p value	% insertion	Driver Gene	p value	% insertion
**MCF7**	KMT2A^**#**^	1.69E-11	0.0173	WISP2	4.94E-10	0
	COMMD3-BMI1	2.33E-09	0.0173*	β-estradiol	1.63E-09	N/A
	ATF4	1.48E-08	0.0173**	ESR1^**#**^	2.01E-09	0
	PHC2	5.19E-08	0.0000	estrogen	6.90E-07	N/A
	MYC^**#**^	8.04E-07	0.0087*	RNF31	1.13E-06	0
**CD34**	CSF3	5.16E-09	0	CD3	1.22E-10	0.0051
	HOXA9^**#**^	6.54E-09	0.0102***	TNF	3.57E-10	0.0000
	IL15	7.23E-08	0	IL15	1.02E-08	0.0000
	androgen	1.36E-07	N/A	TCR	2.50E-08	0.0051
	TGFB1	2.02E-07	0.0102*	GATA2^**#**^	2.68E-08	0.0102***
**K562**	GATA1^**#**^	8.29E-07	0.0334***	TP53^**#**^	6.02E-14	0.00222
	IKZF1^**#**^	1.76E-06	0.0393***	ATF4	6.00E-13	0.00633
	TP53^**#**^	5.93E-06	0.0022	KITLG	2.44E-12	0
	NR3C1	1.09E-05	0.0041	IL3	5.11E-12	0
	FLT1	1.38E-05	0.0277***	EIF2AK3	3.20E-11	0.00158
**HepG2**	TGFB1	8.60E-13	0.00765	TP53^**#**^	2.02E-11	0.00191
	β-estradiol	7.28E-11	N/A	MYC^**#**^	3.62E-11	0.00139
	NR1I2	4.69E-09	0.0106***	SOX2^**#**^	4.67E-10	0.00031
	TP53^**#**^	5.74E-09	0.00191	HNF4A	5.20E-09	0.0417***
	FN1	1.37E-07	0.0160***	NRG1^**#**^	3.65E-08	0.00311

Hash symbol (#) represents a known cancer driver gene, while asterisks represent statistical significance with * being p<0.05, ** p<0.01 and *** p<0.001.

This analysis was also carried out for super-enhancer-associated gene sets, taking the top 500 genes as determined by the ‘rank’ on the dbSUPER database entry, or the entire set if there were fewer than 500 genes. Although the super-enhancer-associated gene sets were in some cases smaller (range 98–742 genes), they yielded predicted upstream regulators with similar statistical scores. Again these upstream regulators revealed relatively little overlap between GRIP and super-enhancer regulated genes, illustrating the distinct perspectives on underlying growth programmes uncovered by these approaches. A further difference is that retroviral integration was significantly more likely to target the GRIP upstream regulators compared to the super-enhancer set (p = 0.005, Fisher’s exact test).

## Discussion

**Fig 8:**
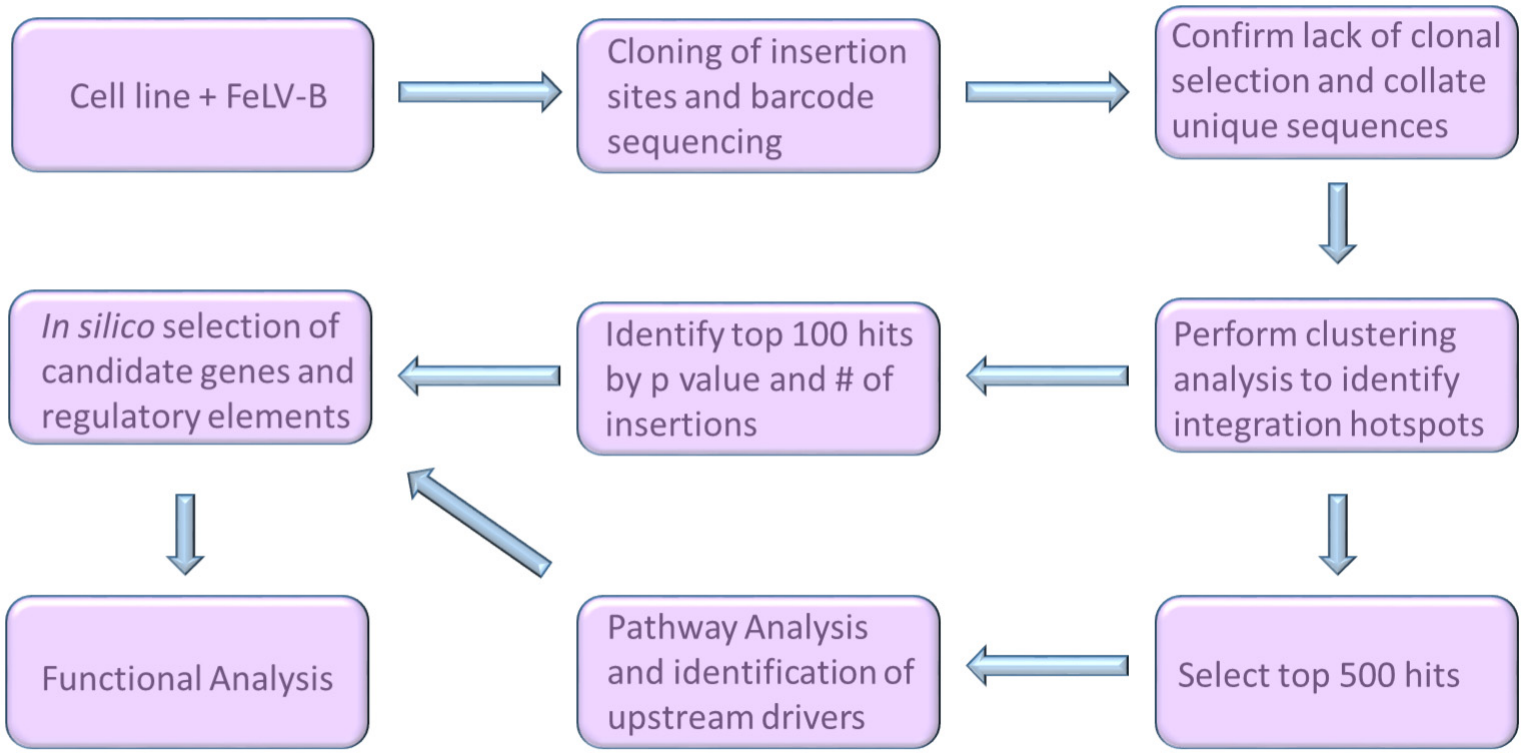
Overview of GRIP. Flow chart showing the experimental workflow, from infecting cells through deep sequencing and the bioinformatics steps necessary to identify candidate genes or regulatory elements.

This study shows that gamma-retrovirus integration in human cells is skewed towards cancer driver genes in a highly cell-type specific manner. While gamma-retroviruses have been widely used as cancer gene discovery tools due to their insertional mutagenic potential in their natural hosts, this approach required the onerous task of collecting multiple end-stage tumours from animal models and comparative genomic analyses to confirm the relevance of findings to human cancer [5–7,11,30]. In contrast, the present study shows that infection and short-term *in vitro* culture followed by deep sequencing of integration sites and computational analysis can be used to mine human cancer cells of many types directly for active gene programmes and potential therapeutic targets. Additionally, the value of this approach is likely to increase over time as knowledge of cancer genes and networks increases and databases are more comprehensively annotated. Fig 8 outlines a protocol for the application of GRIP in future screens for cancer-relevant genes.

Infection of human cells with gamma retroviruses requires the use of amphotropic variants or pseudotypes to overcome the lack of receptor for common strains [17,18]. FeLV-B is a common, naturally occurring amphotropic variant [19] that can be used without modification to infect and integrate into virtually all human cells in culture. Moreover, FeLVs show strong conservation in the C-terminal integrase loop that in MLV has been shown to mediate interaction with BET/Brd, and this study of FeLV integration in MCF-7 breast cancer cells revealed a marked preference for transcriptional start sites and active chromatin marks similar to that previously noted for murine gamma-retroviruses [17,18]. FeLV therefore presents the same complex pattern as MLV with regard to statistical association with multiple chromatin marks, although BET is the only tethering factor that has been directly implicated [13–15]. One possible explanation is that BET interaction is the essential mediator and associations with other chromatin marks are simply due to their physical proximity. In support of this hypothesis, there are many FeLV integration clusters where, in particular, H3K27ac and H3K4me3 marks coincide. Alternatively, clusters of integration at regions of low H3K27ac marking could arise from chromatin looping [31], bringing new target sites into proximity to the tethered provirus. In support of BET interaction as the primary driver of specificity, deletion of the C-terminal MLV integrase loop that targets BET and viral replication in the presence of BET inhibitors both diminish the bias of MLV integration towards promoter/TSSs [13–15].

A meta-analysis was conducted to compare the FeLV GRIP dataset with previous large scale MLV and vector integration sets from normal and malignant human cells. A notable outcome was the selection for cancer genes in all cases, particularly in the top target genes for each cell type. This phenomenon was not fully replicated in the HepG2 dataset, but we note that this was by far the largest dataset where there was evidence of loss of definition due to saturation at the major hotspots. These observations should guide future application of GRIP, as the need for multiple insertions to ensure robust statistics has to be set against the risk of saturation. Meta-analysis also revealed profound cell type specific differences in the top target genes identified by GRIP in the four cell backgrounds, distinguishing these clearly from abundantly expressed ‘house-keeping’ genes. The observed integration site preference of gamma-retroviruses is clearly more selective than a simple preference for open chromatin, suggesting that GRIP will have utility alongside methods such as ATAC-Seq and FAIRE-Seq, where respectively, transposon tagging or cross-linking and fractionation are used to analyse open chromatin regions [32,33]. Another potentially useful feature of GRIP was revealed by *in silico* prediction of upstream regulators using Ingenuity Pathway Analysis, which strongly inferred a series of master oncogenes controlling the growth of the cancer cell lines. Moreover, the upstream regulators were themselves targets for retroviral insertions, showing that these gene loci had significant transcriptional activity in the cancer cells. Although cancer genes also emerged from GRIP targets and upstream regulators in normal CD34+ cells, predicted upstream regulators included cytokines that were not targeted by proviral insertion. While further analyses will be required to address this question, an interesting possibility is that GRIP in combination with upstream regulator prediction may be capable of discriminating normal cell growth pathways controlled by exogenous growth factors and cancer cell growth orchestrated by internal, constitutively active programmes.

However, not all GRIP targets were cell-type specific and notable exceptions included the long non-coding RNA *MALAT1*, with up to 0.17% of all gamma-retrovirus insertions mapping in or near this element. There is a bourgeoning literature on the role of *MALAT1* over-expression in human cancer (reviewed in [34]). Paradoxically, *Malat1* appears to be dispensable for mouse development, while depletion has only modest effects limited mainly to nearby genes [35]. It is unclear whether the murine gene is also targeted by gamma retroviruses as there are relatively few MLV insertions at *Malat1* in the retroviral tagged gene database (http://variation.osu.edu/rtcgd/index.html) or in progressing lymphomas analysed by deep sequencing [22], suggesting that clonal expansion is not a common consequence of MLV insertions at this site in the mouse. It will be important to establish whether this reflects a species difference and whether clonal expansion follows insertions at *MALAT1* e.g. in human-mouse cancer xenografts adventitiously infected *in vivo* by murine gamma-retroviruses [24].

The predilection of gRV integration for cancer genes is also relevant to viral biology. The ability to ‘seek out’ active chromatin ensures a favourable environment for proviral transcription. Moreover, by selectively targeting cancer drivers and growth effectors, an additional advantage may be gained by promoting the proliferation and/or survival of the infected cell. GRIP carried out early after infection allows the broad spectrum of preferred integration domains sites to be sampled, while continued cell passage would be expected to select for the most efficient mutagenic events. In MCF-7 cells we found no evidence for significant clonal selection due to proliferation or enhanced survival, as we observed no proviral orientation bias or increased copy number of insertions at the most highly targeted genes. However, we cannot exclude the possibility that insertions that have a strongly deleterious effect are counter-selected even in short-term culture. In this respect it is interesting to consider that gamma retroviral preference for cancer genes is operative in multiple cell types, while these viruses are prone to induce mainly haematopoietic malignancies in their natural hosts [11,36] A likely explanation is provided by the gamma retroviral LTR enhancer elements that are critical for efficient insertional mutagenesis and are selectively active in specific cell types [37]. The ability of retroviral vector integration to initiate clonal expansion resulting in patient leukaemias with insertions at a subset of integration hotspots (*LMO2*, *BMI1*, *CCND2*) in gene therapy trials indicates the translational relevance of these observations [4,38,39]. This propensity to drive cell expansion is not a barrier to the use of GRIP in haematopoietic cancers but highlights the need to ensure that an early pre-growth selection sample is analysed.

The term super-enhancer was coined to denote a large cluster of enhancers that displays dense binding of master regulators and plays a role in tissue-specific cell identity [25]. The clarity of distinction between super-enhancers and conventional enhancers has been questioned [29], but the concept has been widely adopted, and is relevant to this GRIP study as H3K27ac appears to be one of the defining histone modifications and BET/bromodomain factors are among the abundant regulators associated with these chromatin features [26]. Moreover, inhibitors of BET binding can disrupt the growth of cancer cells and this phenomenon has been attributed to the sensitivity of super-enhancers, driving oncogenes such as MYC and BCL2, to BET disruption [26,27]. The assumption that known players are involved is attractive, although the cell type specificity of super-enhancers remains puzzling in light of the widespread sensitivity to, and apparently cancer-specific action of, BET inhibitors.

It was therefore of great interest to compare the loci marked by GRIP with databases of super-enhancers identified in the same cell lines or cell types. While we found that super-enhancers and GRIP targets had similar levels of enrichment for cancer driver genes, there was relatively little overlap between the genes identified, and between GRIP and super-enhancer top hits generally. This is perhaps not surprising, as GRIP strongly targets TSSs as well as distal enhancers. However, there was also a significantly greater chance of marking the putative upstream regulators of GRIP targets by proviral insertion than for the upstream regulators predicted by the super-enhancer set. This finding implies that the datasets are non-redundant and it is conceivable that the ability of GRIP to target promoters as well as enhancers provides a distinctive and possibly unique window on the regulatory circuits in operation. We further hypothesise that the gamma-retroviral integration machinery recognises an as yet undefined chromatin state that is highly enriched for cell type-specific, cancer-relevant genes. As databases such as IPA grow in information content, it is likely that the utility of such analyses for the identification of the key drivers will increase.

Overall, GRIP is a novel and potentially powerful technique that can add significantly to existing approaches to the elucidation of driver genes in human cancers.

## Materials and Methods

### Viral Infection

MCF-7 cells were a gift from K. Blyth (Beatson Institute, Glasgow, UK) and cultured in MEM (10370047 Gibco, Life Technologies, Paisley, UK) containing 10% FCS (HyClone, Perbio, Life Technologies, Paisley, UK), 100 μg/ml Penicillin/Streptomycin (15140 Gibco), and 2nM Glutamine (25030 Gibco). Additionally, 0.01mg/ml Insulin (I9278 Sigma, Munich, Germany) and 1mM Sodium pyruvate (11360 Gibco) were used to supplement media. Cells were infected with 0.45μm-filtered supernatant harvested from sub-confluent FeLV-B-infected AH927 cells (feline fibroblasts) at a multiplicity of infection of 1. The medium was replaced after 2 hours of infection, and cells cultured for a further 14 days.

### Sequencing of DNA

DNA was extracted from cells using a DNeasy kit (Qiagen, Hilden, Germany) according to the manufacturer’s instructions. Genomic DNA was sheared down to average size of 400bp using Covaris-S2 (CovarisMA, USA), and end repair performed using NEB end repair mix (New England Biolabs, MA, USA).

Double stranded DNA adaptors Linker-1 (GCTTGATCGAGCAGTTGACCCGGGAGATCTGtcttatAATTC) and Linker-2 (PO4-GAATTataagaCAGATCTCCC-NH2) were ligated with unique barcodes (underlined lowercase region, to avoid cross contamination among samples) onto the DNA. The ligation products were then amplified using the FeLV LTR Outer Primer (CAAGTCTTTGTTGAGACTTGAC) and the Linker Outer Primer (GCTTGATCGAGCAGTTGACC). Nested PCR was then performed with the FeLV-LTR Inner Primer (GTACCCGTGTACGAATAAAGC) and the Linker Inner Primer (AGTTGACCCGGGAGATCTG). LM-PCR products were then built into an Illumina library using the NEBNext library construction kit (New England Biolabs) and sequenced on an Illumina MiSeq platform (Illumina, CA, USA) to generate pair end reads with length of 2 x 250bp.

Data pre-processing and sequence alignment to the hg19 assembly of the human genome was performed as described previously [40]. .bed files of insertion data are attached as S1 Dataset.

### Microarray Analysis

Uninfected MCF7 cells were grown to give 3 independent biological replicates and RNA extracted as reported previously [24]. Microarray analysis was performed with GeneChip Human Gene 2.0 ST arrays (Affymetrix, High Wycombe, UK) by the commercial provider ATLAS Biolabs according to standard protocols (Berlin, Germany). Affymetrix Expression Console software with RMA normalisation was used to generate .chp files, and then CLC Genomics Workbench software was used to perform statistical analysis, including fold changes, p-value statistical significance and False Discovery Rate (FDR) multiple testing correction.

### Bioinformatic and statistical analyses

#### Nearest gene analysis

Insertions were grouped by proximity to their nearest gene, using a 25kb window upstream and downstream of genes of interest, thus incorporating local regulatory elements. For each gene, enrichment of insertions in the window was calculated using a hypergeometric test. The mappable genome was estimated as the union of all gene windows. FDR correction was applied to address multiple testing [41]. Refseq gene definition was used (http://genome.ucsc.edu; accessed 9 Jan 2015).

#### Analysis of insertions relative to transcription start sites (TSSs)

MCF7 insertions were uploaded as a .bed file into RStudio and then analysed using the Bioconductor package ‘ChippeakAnno’ for proximity to transcriptional start site (TSS) and other features as described [42]. Insertions were annotated using the TSS.human.GRCh37 dataset, which describes the hg19 assembly.

#### Analysis of proviral orientation bias

Assessment of orientation bias was performed as described [22] with the following modification. CIS in this case were defined as the clusters of insertions near to a gene, as defined by the nearest gene analysis (above), including the 25kb either side of the gene to give the same window as the nearest gene analysis. The proportion of insertions in the forward or reverse orientation was compared to that in the genome as a whole, and the likelihood of this occurring by chance determined by Fisher’s Exact test with Bonferroni or Benjamini-Hockberg correction for multiple testing.

#### Global analysis of histone modifications

All available histone modification data for MCF7 was imported into RStudio from the ENCODE ChIP repository using the AnnotationHub package from Bioconductor (Morgan M, Carlson M, Tenebaum D and Arora S. https://bioconductor.org/packages/release/bioc/html/AnnotationHub.html). In the case of H3K27ac, H3K9me3, H3K27me3 and H3K36me3 narrow peak files were all that was available. In the case of H3K4me3 both broadpeak and narrowpeak files were available, however in this case broadpeak annotation was used as the spread of peak widths was more similar to the narrowpeak widths of the other files. All peaks for all histone marks, as well as MCF7 insertion site data, were annotated using TSS.human.GRCh37, and gene IDs added using the ‘org.Hs.eg.db’ dataset. Annotated ChIP and MCF7 insertion data were then converted to GRanges objects and overlap assessed using the ‘makeVennDiagram’ function from the ChippeakAnno package. P values (from hypergeometric testing) and overlap counts were exported as separate dataframes. The same process was followed for K562 and HepG2 cells. In the case of CD34 primary cells, data for patient RO_01536 were obtained from the Human Epigenetic Roadmap repository, accessed via AnnotationHub, and the same analysis applied.

#### Analysis of overlap of most significant ChIP targets and insertion targets

Raw data files for histone modifications in MCF7 cells were obtained from the ENCODE repository as either .bam files (where available) or .fastq files (for H3K4me3). Preprocessing was performed using the Galaxy Platform. Where .fastq files were used as a starting point, data were screened using FastQC and then trimmed as appropriate using FastQTrimmer. Data were aligned to the hg19 assembly of the human genome using Bowtie2 to obtain .bam files. ChIP peaks were then called using MACS with the Model Fold (MF) parameter set to 10, and peaks annotated with gene names using the Galaxy/Cistrome platform with the peak2gene algorithm. The top 500 hits were selected by pscore; where this was tied, all peaks with that score were included and the difference in sample size accounted for statistically.

Overlap between the top ChIP peaks and the top MCF7 insertion target sites was determined using custom R scripts. The likelihood of such overlap occurring by chance was then determined by Monte-Carlo simulation that randomly sampled the full Refseq gene list for a given sample size and then calculated the overlap with the top hits list. 1x10^6^ simulations were done for H3K4me3, H3K9me3, H3K36me3 and H3K27me3. 60 x 10^6^ simulations were done for H3K27ac, after which still no overlap of the size observed occurred by chance.

#### Comparison of top hits with highly expressed genes

Expression data from MCF7 cells was obtained from Microarray analysis, and ranked according to average intensity in cells in the basal state. Custom R code was written to then take equal sized samples (50, 100 genes etc) of top GRIP targets and most highly expressed genes, and assess the level of overlap. The level expected by chance was determined using Monte-Carlo simulation as described above, and p values calculated.

#### Pathway analysis

Ontological analysis was performed on gene lists using commercially available Ingenuity Pathway Analysis (IPA) software (IPA, QIAGEN Redwood City, www.qiagen.com/Ingenuity). Pathway annotation and upstream regulator analysis were exported and summarised.

### Statistical methods

Significance of enrichment of cancer driver genes compared to the genome as a whole was established using Chi squared tests. The significance of copy number difference between top 100 genes and the genome as a whole was established using an unpaired two-tailed Student’s t-test with unequal variance. The significance of difference between the number of retroviral targets in predicted upstream transcriptional regulators in GRIP compared to super-enhancer gene sets was calculated considering all of the gene upstream regulators for all cell lines and excluding small molecule regulators as these cannot be targeted by retrovirus. The total number of upstream regulators for each of the GRIP and super-enhancer datasets was 18, of which 10 were integration targets with GRIP and 2 were integration targets in the super-enhancer set. A comparison of total integration targets versus non integration targets for GRIP (10 vs 8) and the super-enhancer set (2 vs 16) was performed using Fisher’s exact test in order to obtain a p value.

## Supporting Information

S1 Dataset FeLV-B insertions in MCF-7 cells mapped to the hg19 build of the human genome. (BED)

S1 Fig UCSC tracks of example FeLV target genes in MCF-7 cells, showing all histone ChIP-seq annotation data that is available. Insertions are shown in purple at the top, with gene structure and then ChIP-seq signal tracks below.
(TIF)

S1 Table Top 100 insertion sites from nearest gene analysis in MCF-7 cells showing gene location, number and position of insertions in the cluster, and statistical significance. Genes that are known cancer drivers are highlighted in green.
(XLSX)

S2 Table Lists of cancer driver genes found in the top 100 gene clusters for MCF-7, CD34, K562 and HepG2 cells. Shown are lists for the GRIP technique, and those from data found in the dbSuper superenhancer database.
(TIF)
