# Supplementary figures and images for: Gamma-Retrovirus Integration Marks Cell Type-Specific Cancer Genes: A Novel Profiling Tool in Cancer Genomics
Source: PLoS One. 2016 Apr 20;11(4):e0154070. doi: 10.1371/journal.pone.0154070 (PMC4838236; doi:10.1371/journal.pone.0154070)

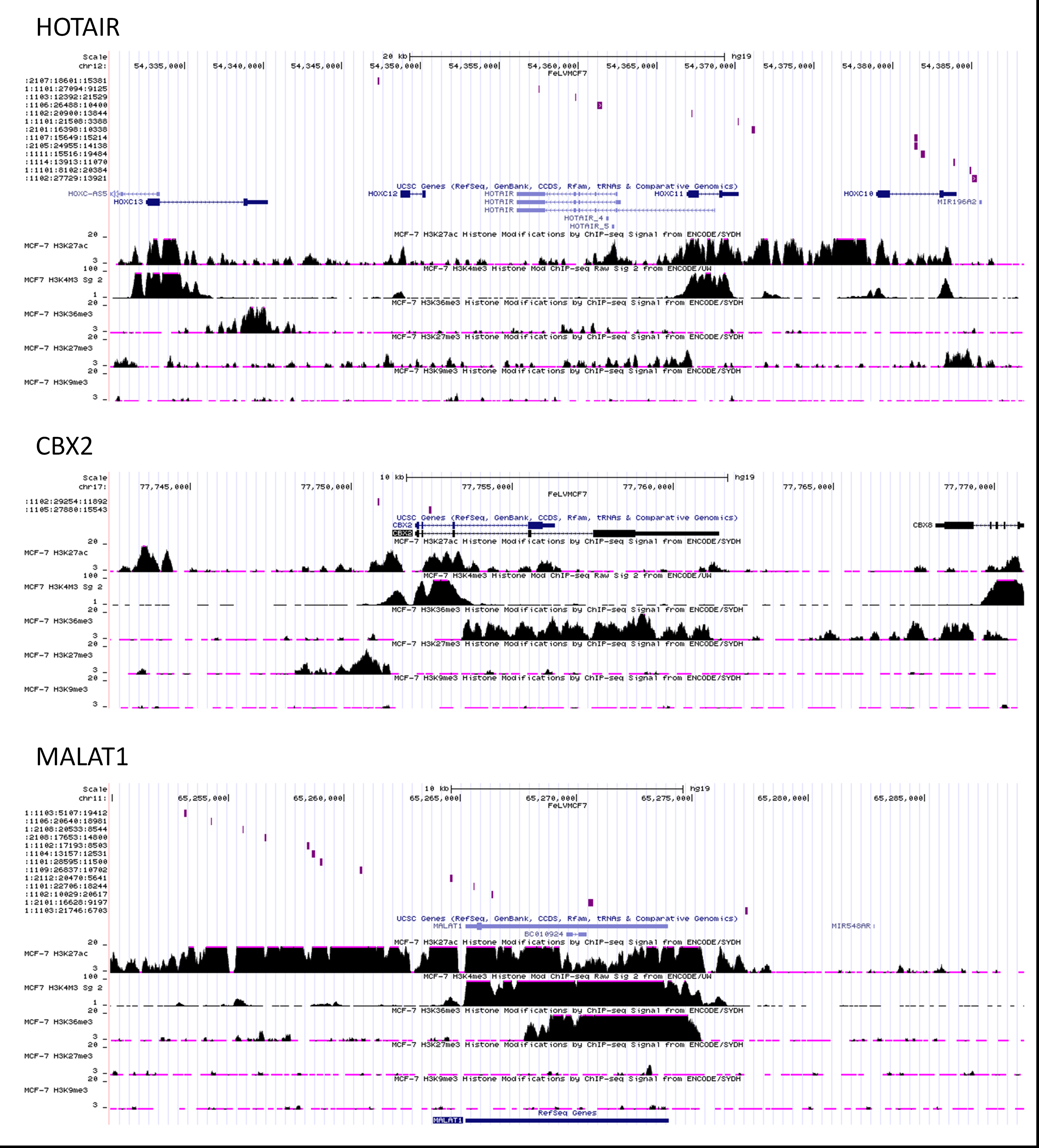

Supplement: S1 Fig — Insertions are shown in purple at the top, with gene structure and then ChIP-seq signal tracks below. (TIF) [file pone.0154070.s002.tif]

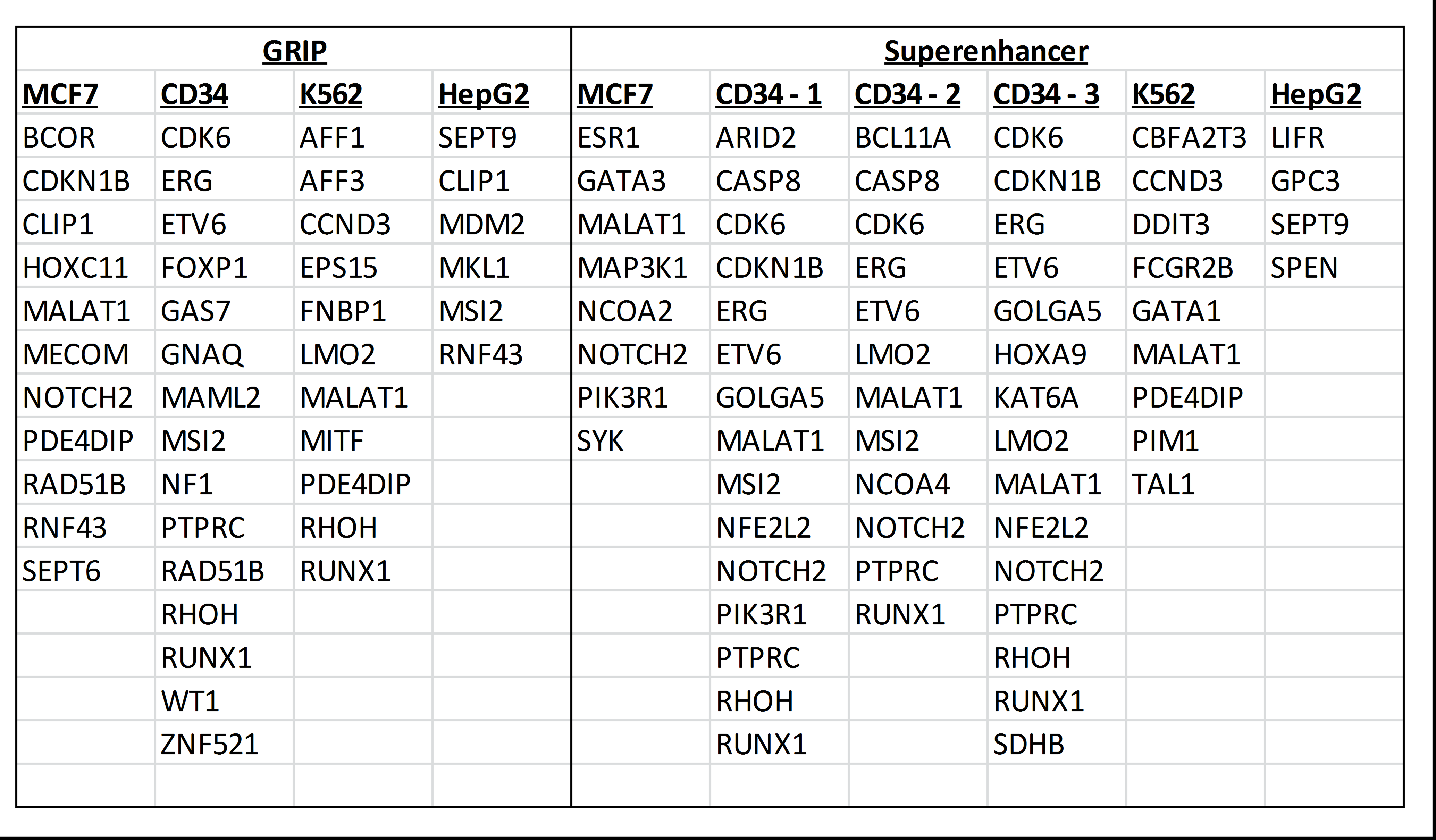

Supplement: S2 Table — Shown are lists for the GRIP technique, and those from data found in the dbSuper superenhancer database. (TIF) [file pone.0154070.s004.tif]
